# Comparison of *Lactuca sativa* growth performance in conventional and RAS-based hydroponic systems

**DOI:** 10.1007/s10499-018-0293-8

**Published:** 2018-08-10

**Authors:** Simon Goddek, Tycho Vermeulen

**Affiliations:** 1 Biobased Chemistry & Technology, Wageningen University & Research, P.O. Box 17, 6700 AA Wageningen, The Netherlands; 2 Greenhouse Horticulture, Wageningen University & Research, P.O. Box 20, 2665 ZG Bleiswijk, The Netherlands

**Keywords:** Aquaponics, Hydroponics, Integrated aquaculture, Horticulture, Decoupled aquaponics, Salt accumulation

## Abstract

A recent study related to aquaponics has shown that hydroponic lettuce grown in aquaculture-derived supplemented water grew significantly better than lettuce grown in a conventional hydroponic system. The principal objective of this study was to verify this finding in a larger setup. Even though the aquaculture water that was added to the aquaculture-based hydroponic system contained relatively high amounts of sodium, we were still able to observe an enhanced growth performance of the lettuce in that system compared to the lettuce grown in the conventional hydroponic nutrient solution. The lettuce final fresh weight was 7.9%, and its final dry weight even 33.2% higher than the one of the hydroponic control.

## Introduction

Aquaponics has received broad attention in both the civil society and the scientific community. Traditionally, aquaponics is an integrated multi-trophic food production system that combines the elements of recirculating aquaculture system (RAS) and hydroponics in one-loop recirculating systems (Knaus and Palm 2017; Schmautz et al. 2016; Yogev et al. 2016). Data from several studies have also showed that aquaponic systems perform well in both water and resource usage (Reyes Lastiri et al. 2016; Suhl et al. 2016). Its lack of the ability to provide optimal conditions for both fish and plants is most likely the main reason why it is currently mainly being used for educational purposes (Villarroel et al. 2016). This is because one-loop aquaponic systems currently cannot compete with yields found in conventional hydroponic systems (Delaide et al. 2016). The commercial breakthrough is still outstanding (dos Santos 2016; Goddek et al. 2015). Recent trends in aquaponics have led to a renewed approach that aims at optimising the conditions in both system components. Kloas et al. (2015) and Goddek et al. (2016) both showed that nutrient concentrations in one-loop systems are suboptimal and suggest to focus on so-called decoupled multi-loop systems to allow optimal conditions (i.e. in nutrient concentrations, temperature, pH, etc.) for both fish and plants. This paper will primarily focus on such multi-loop aquaponic systems.

Although most of the turnover in aquaponic systems is made with the sale of plants (i.e. the sale of the fish only represents a minor proportion of the turnover), only two studies have attempted to investigate the plant growth potential in aquaponic systems on commercial hydroponic nutrient levels. Delaide et al. (2016) claimed that aquaponic-grown lettuce in significantly similar chemical nutrient solutions shows a growth advantage of approximately 40% over hydroponics. Contrary to that, another recent study (Suhl et al. 2016) that investigated tomato growth under similar conditions failed to show any significant production advantage. Taken together, there is a lack of formal experimental data with respect to growth of plants in RAS-based nutrient solutions on hydroponic levels. Consequently, this study was set out to confirm or disaffirm the findings of Delaide et al. (2016) in a larger setup using RAS water versus a typical hydroponic reference.

## Materials and methods

### Experimental setup

Two nutrient flow technique (NFT) systems were placed in a plastic tunnel greenhouse in Bleiswijk, The Netherlands, during a warm August–October season. The gullies of the NFT systems, each system servicing 16 gullies of 7.7 m long, were mounted in two blocks of eight rows and distributed in alternation. The system of cross-over NFT was applied, leading to a drip irrigation nozzle for every plant, while drain water was directly collected and did not affect neighbouring crops. These individual water nozzles gave a water flow of 2 L per hour. No additional CO_2_ was applied. The recirculation container of each NFT system contained 250 L of water.

**Fig. 1:**
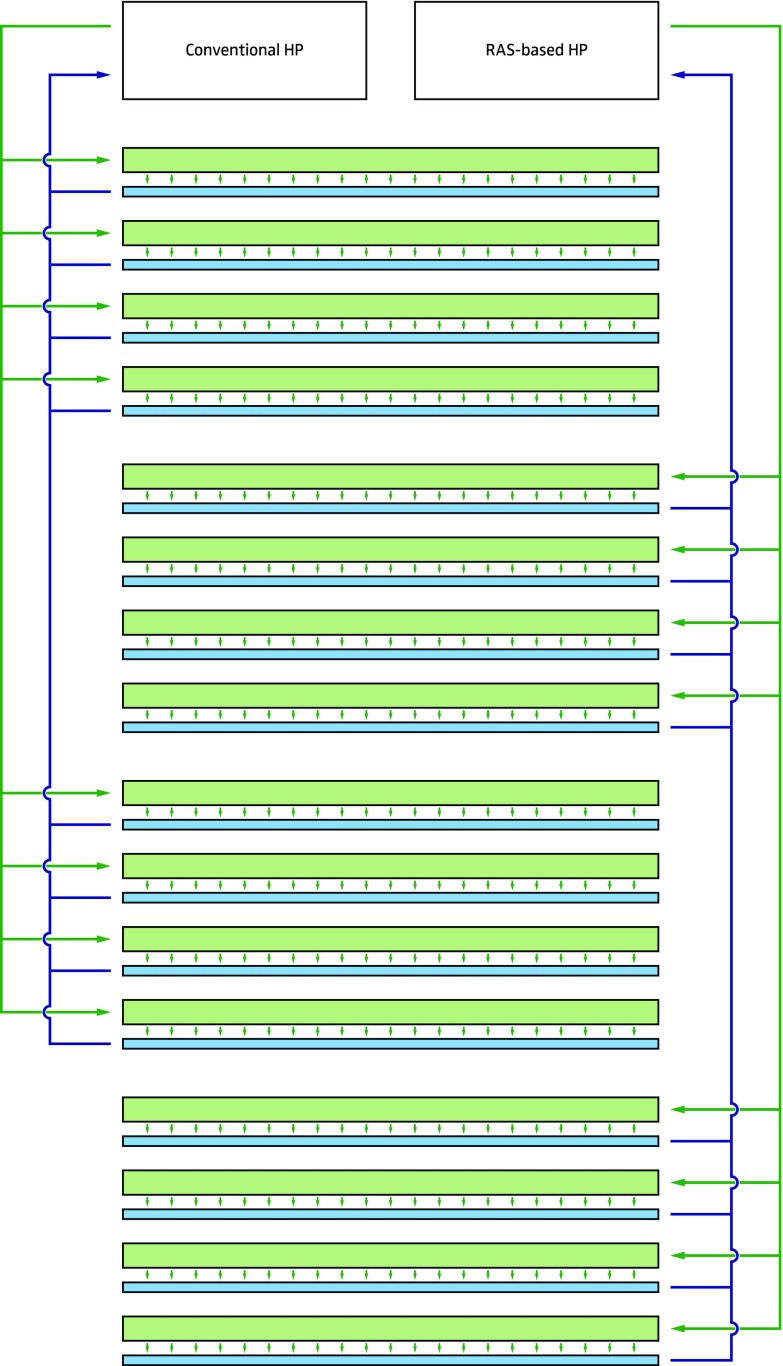
Scheme of the experimental setup consisting of a conventional hydroponic system as well as a RAS-based hydroponic system. Every plant was drip-irrigated individually. The drain water was led back to the respective recirculation containers via a shaded gutter

**Fig. 2:**
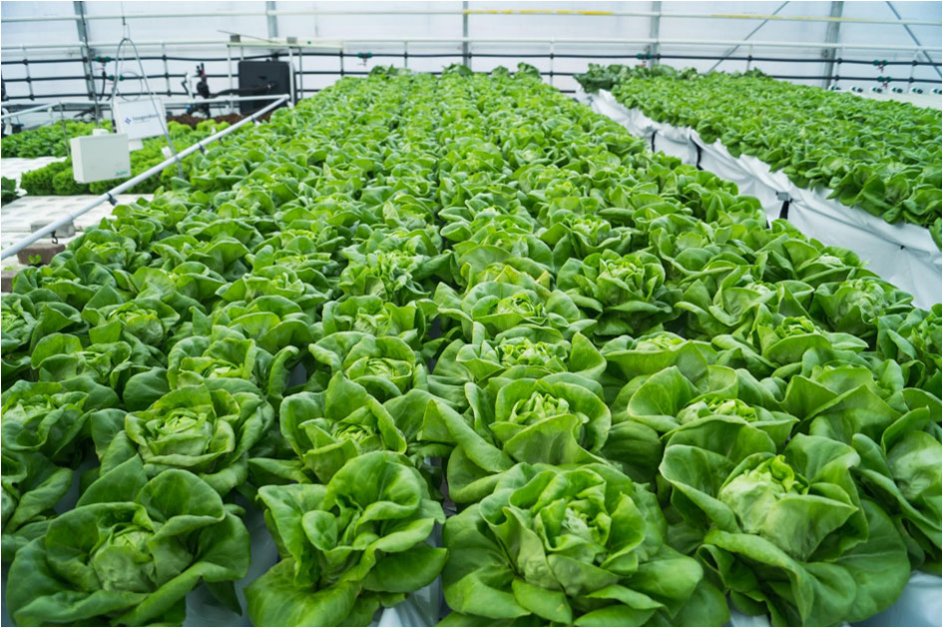
Picture of the experimental setup in Bleiswijk, The Netherlands

Gullies were planted with 38 lettuces each, leading to a planting density of 12 heads per square meter. Hydrologically speaking, this approach, however, cannot be considered as a repetition. The scheme as well as a picture of the experimental setup can be seen in Figs. 1 and 2.

The hydroponic treatment tank has been filled up with rain water continuously and the RAS treatment tank with 30% RAS water and 70% rain water. To both tanks, hydroponic nutrient solutions (General Hydroponics, FloraMicro, FloraGrow and FloraBloom, 3:2:1 mixing ratio) were added daily. The RAS water was taken from a RAS system cultivating carps just hours before it was added to the hydroponic system. The climate was monitored (temperature, relative humidity (RH), irradiation). Water loss due to evapotranspiration and leakage was replaced continuously in the basin, while the electrical conductivity (EC) and acidity (pH) were measured daily, and kept constant on 1800 μS cm^−2^ and pH 5.0–6.0 respectively. Every 2 weeks, 40 L RAS water was added to the hydroponic sump.

### Water analysis

Once every 2 weeks, water of both systems, as well as the RAS water from an experimental carp RAS system in Wageningen, has been sent to the lab for analysis of the plant-relevant ion composition. The water samples have been measured by the commercial lab Groen Agro Control, Delft, The Netherlands, using HPLC equipment according to the ISO 17025 norm.

### Lettuce

Butterhead lettuce (variety Cosmopolia, RZ) were sown in 4 × 4 peat blocks and put directly into the system on the 23rd of August 2016. Seven weeks after planting (11th of October), 20 lettuce shoots were randomly selected, harvested and weighed individually. Prior to sending in the milled lettuce shoots for lead analysis, the lettuce heads of each system were cut into small pieces, weighed and merged in brown paper bags (40 × 20 × 20 cm) and dried (for 24 h at 103 °C) to determine their dry weight. The leaf analysis of the nutrients was performed with an ICP-OES by Groen Agro Control according to their certified analysis protocol.

### Statistical analysis

Data are presented as mean *±* standard deviation (SD) and ranges respectively of *n* samples. Analysis of statistical significance and ANOVA were conducted in R (R Core Team 2013). Furthermore, the nonparametric two-sample Kolmogorov–Smirnov test was used to test whether the two (i.e. in the RAS and HP system) Na concentration probability distributions differ. Genstat software was used to conduct a principal component analysis with respect to the lettuce’s nutrient composition.

## Results and discussion

**Fig. 3:**
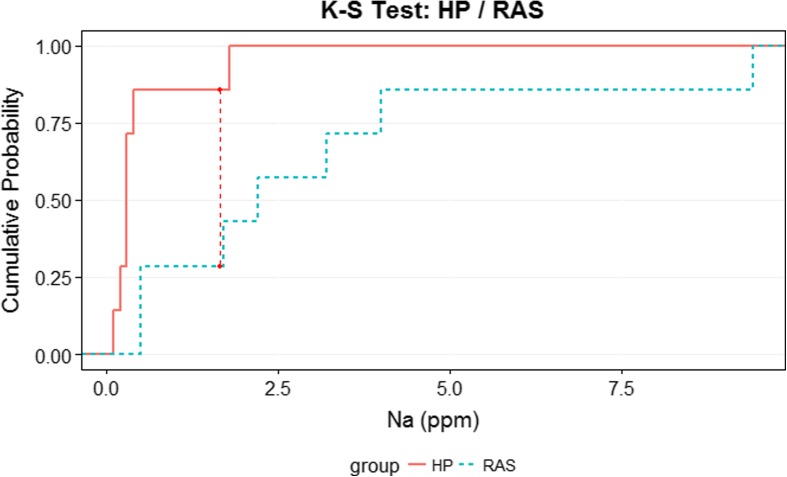
Two-sample Kolmogorov–Smirnov test, where *D* = 0.857, and *p* = 0.01. Thus, it is reasonable to assume that the data comes from different distributions, since *p* > 0.05

The EC was maintained constant throughout the experiment. Average day temperatures were 17.7 °C, while average outside irradiation was 1584.66 J cm^−2^ day^−1^ and light transmission index of the foil greenhouse was 57.7%. The nutrient concentrations of the RAS water and the respective systems can be seen in Tables 1 and 2. What attracts attention is that the difference in sodium concentrations is significant (Table 1, Fig. 3). This difference can be attributed to the RAS water (Table 1). In RAS systems, sodium chloride is often added to counteract stress, restore osmoregulation and prevent and control diseases. Adding sodium chloride in aquaponic systems, however, is not common practice, as it is known to inhibit plant growth.

**Table 1:** RAS water composition (mean *±* SD, *α* = 0.05, *n* = 7)

pH	EC	K^+^	Na+	Ca^2+^	Mg^2+^	NO_3_^−^	Cl^−^	SO_4_^2−^	HCO_3_^−^	P	Zn^2+^
–	mS cm^−1^	mmol L^−1^	mmol L^−1^	mmol L^−1^	mmol L^−1^	mmol L^−1^	mmol L^−1^	mmol L^−1^	mmol L^−1^	mmol L^−1^	μmol L^−1^
7.66 ± 0.11	1.18 ± 0.26	0.20 ± 0.28	8.62 ± 2.37	0.72 ± 0.08	0.12 ± 0.04	1.58 ± 0.38	7.44 ± 0.38	0.22 ± 0.08	1.06 ± 0.38	0.04 ± 0.05	0.16 ± 0.05

**Table 2:** Nutrient concentration in the water of each system (mean *±* SD, *α* = 0.05, *n* = 7)

Parameter	Unit	System HP (mean ± SD)	System RAS (mean ± SD)	ANOVA
pH		5.10 ± 0.70	5.86 ± 0.91	*p* = 0.105
NH_4_^+^	mmol L^−1^	1.56 ± 0.92	0.87 ± 0.74	*p* = 0.151
K^+^	mmol L^−1^	3.39 ± 2.10	2.36 ± 2.00	*p* = 0.367
Na^+^	mmol L^−1^	0.49 ± 0.59	3.07 ± 3.08	*p = *0.049
Ca^2+^	mmol L^−1^	2.16 ± 0.46	2.24 ± 0.53	*p* = 0.753
Mg^2+^	mmol L^−1^	0.15 ± 0.39	0.14 ± 0.36	*p* = 0.728
Fe^2+^	μmol L^−1^	53.74 ± 37.81	39.60 ± 23.19	*p* = 0.415
Mn^2+^	μmol L^−1^	17.31 ± 9.84	12.99 ± 9.57	*p* = 0.420
Zn^2+^	μmol L^−1^	73.76 ± 55.06	29.01 ± 22.58	*p* = 0.070
Cu^2+^	μmol L^−1^	2.63 ± 1.03	2.14 ± 1.09	*p* = 0.409
Mo^6+^	μmol L^−1^	0.66 ± 0.56	0.47 ± 0.53	*p* = 0.537
P	mmol L^−1^	0.97 ± 0.49	0.94 ± 0.47	*p* = 0.913
B (III)	μmol L^−1^	19.00 ± 6.00	19.14 ± 5.52	*p* = 0.964
HCO_3_^−^	mmol L^−1^	0.06 ± 0.10	0.37 ± 0.47	*p* = 0.110
NO_3_^−^	mmol L^−1^	8.07 ± 3.13	6.63 ± 2.76	*p* = 0.378
Cl^−^	mmol L^−1^	0.79 ± 0.50	2.84 ± 2.67	*p = *0.068
SO_4_^2−^	mmol L^−1^	1.17 ± 0.35	1.09 ± 0.39	*p* = 0.650

**Fig. 4:**
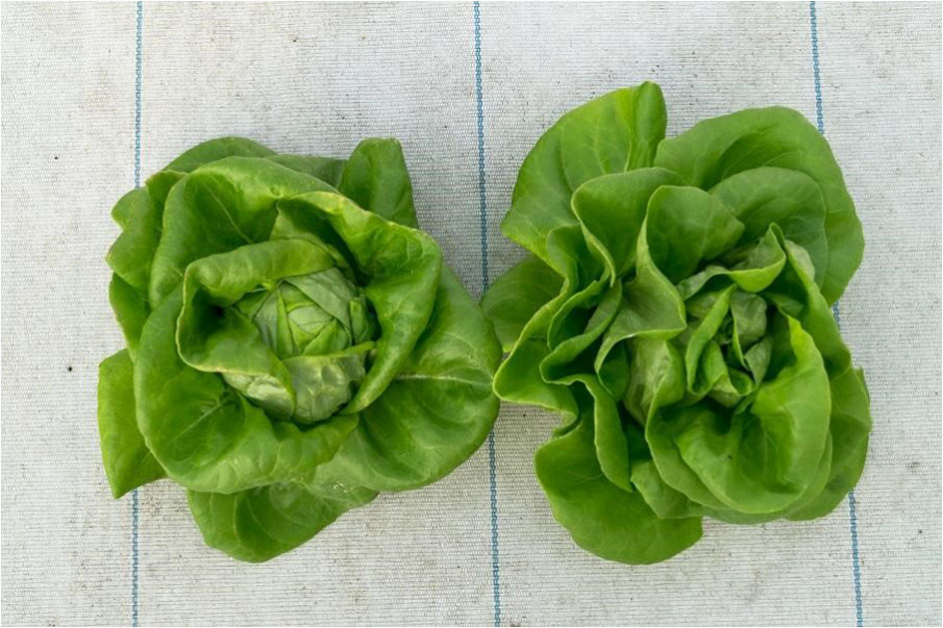
Picture of a RAS-system lettuce (left) and a purely hydroponically grown lettuce (right). This picture was taken after harvesting the crops

Throughout the experiment, a visually different growth was observed. At harvest, the lettuce from the RAS water system was further developed than the lettuce that was grown in pure hydroponic water. This was indicated by the fact that the RAS-system lettuce was in the heading stage at harvest, while the hydroponic-system lettuce was still in the previous cupping stage (Fig. 4). Table 3, which shows the fresh weight of the plants, rejects the possible assumption that the faster development could have been caused by physiological stress due to high sodium chloride values. Although lettuce is known to be a relatively sodium-insensitive plant, it is remarkable to see that the fresh weight difference is highly significant to the advantage of the RAS-system lettuce. Its final fresh weight was 7.9%, and its final dry weight even 33.2% higher than the one of the hydroponic control. It is also worth mentioning that the dry matter (%) of the lettuce grown in the RAS was 2.5 times higher (7.8%) than the lettuce grown in the pure hydroponic environment (3.1%). While in our case the chemical composition of the RAS water was suboptimal for plant production, the findings are still consistent with those of Delaide et al. (2016), even though our observations in final weight differences were lower.

**Table 3:** Fresh weight (mean *±* SD, *α* = 0.05, *n* = 20) and dry weight (mean, range […, …], *n* = 2) of the lettuce

Parameter	Unit	System HP (mean ± SD) and [range]	System RAS (mean ± SD) and [range]	ANOVA
Wet weight	g	286.83 ± 47.78	309.48 ± 32.96	*p* = 0.016
Dry weight	g	9.08 [7.92, 10.24]	12.09 [10.40, 13.79]	*p* = 0.280

**Fig. 5:**
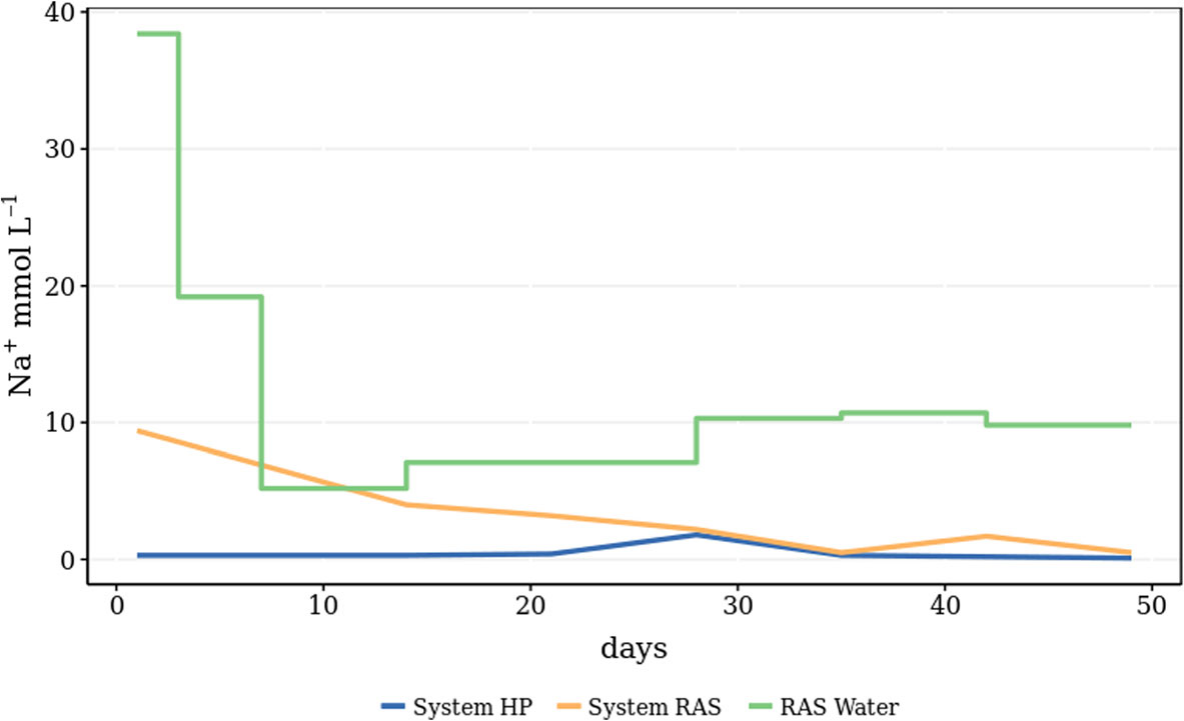
Sodium concentration in both RAS and HP systems (yellow and blue line respectively) as well as the sodium concentration of the RAS water (green line) that was added to the RAS system on a weekly basis. System RAS was first prepared with RAS water of higher sodium concentration (data not shown)

**Fig. 6:**
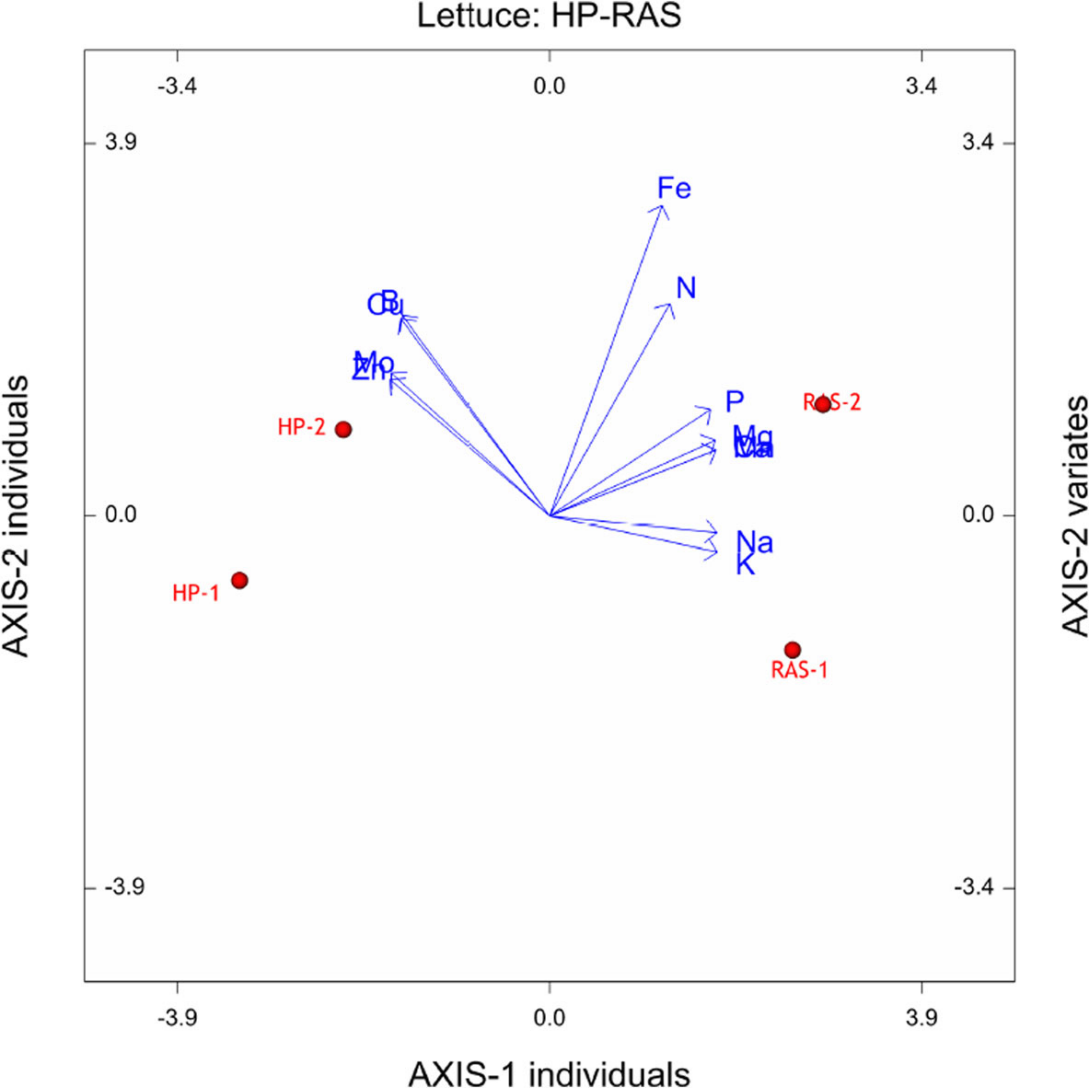
A principal component analysis of the lettuce leaf composition (Table 4)

Despite repetitive addition of sodium-rich RAS water, the treatment did not show accumulation to the extent expected (Fig. 5). This could be due to solely increased uptake of sodium by the plants. Based on modelled evapotranspiration by the plants (i.e. Penman Monteith Equation), calculated uptake concentrations were found to be 0.04 and 0.21 mmol L^−1^ for the control and RAS treatment respectively. Leakage of the system, combined with continuous refill of the water reservoir with rain water, would explain the decrease of sodium concentration over time. EC levels remained the same due to daily application of nutrient solution. These concentrations are still lower than uptake concentrations of 0.5–0.8 mmol L^−1^ found under high sodium levels in the nutrient solution (Sonneveld and Voogt 2009). Taking a look at the lettuce leaf composition (Table 4) shows that the uptake of the other salts K, Ca, Mg, and P also was stimulated in the RAS-water growth environment (Fig. 6), while the uptake of the micronutrients Zn and Mo was rather inhibited compared with the lettuce grown in the hydroponic environment. In the same figure, it can be seen that the uptake of the macronutrients P, Mg, Ca, Na and K correlates with the RAS-system-grown lettuces, and the micronutrients Cu, B, Mo and Zn correlate with the HP-system-grown lettuces. Fe and N rather seem to be independent variables. It could be hypothesised that a possible interaction with micro-organism in the rhizosphere caused a better photosynthesis and nutrient uptake. However, we do not know what the uptake rate of the hydroponic control would be if the same amount of Na was added there. Consequently, it is yet to find out which plant species are able to take up these high amounts of sodium and to what degree different species would have to be combined in one system to avoid accumulations.

**Table 4:** Lettuce leaf composition of the dry matter (dm) (range […, …] samples, *n* = 2)

Parameter	Unit	System HP	System RAS	ANOVA
K^+^	mmol kg^−1^ dm^−1^	1808.00 [1795, 1821]	2327.50 [2325, 2330]	*p* = 0.000
Na^+^	mmol kg^−1^ dm^−1^	25.50 [25, 26]	91.90 [86.1, 97.7]	*p* = 0.008
Ca^2+^	mmol kg^−1^ dm^−1^	325.00 [314, 336]	408.50 [396, 421]	*p* = 0.038
Mg^2+^	mmol kg^−1^ dm^−1^	167.00 [160, 174]	206.50 ± 7.78 [201, 212]	*p* = 0.047
N	mmol kg^−1^ dm^−1^	3708.00 [3526, 3890]	3909.50 [3864, 3955]	*p* = 0.395
P	mmol kg^−1^ dm^−1^	311.00 [306, 316]	369.00 [353, 385]	*p* = 0.075
Fe^2+^	mmol kg^−1^ dm^−1^	3.25 [3.1, 3.4]	3.70 [3.3, 4.1]	*p* = 0.403
Mn^2+^	mmol kg^−1^ dm^−1^	3.55 [3.4, 3.7]	4.80 [4.6, 5.0]	*p* = 0.038
Zn^2+^	mmol kg^−1^ dm^−1^	15.50 [15, 16]	7.35 [6.5, 8.2]	*p* = 0.014
B	mmol kg^−1^ dm^−1^	3.20 [3.2, 3.2]	3.05 [3.0, 3.1]	*p* = 0.095
Mo^6+^	μmol kg^−1^ dm^−1^	25.20 [24.6, 25.8]	18.10 [17.2, 19.0]	*p* = 0.022
Cu^2+^	μmol kg^−1^ dm^−1^	170.50 [170, 171]	148.50 [142, 155]	*p* = 0.078

In addition, Fig. 4 shows the harvested crops. It can clearly be seen that the leaves of the RAS-system lettuce overlap and cover the growing point of the plant, which indicated that the lettuce is being in the heading stage, while the hydroponic system lettuce is still in the cupping stage. The cupping stage is prior to the heading stage, where the inner leaves begin to curl inwards on the edges.

## Conclusion

The purpose of the this study was to test the hypothesis that lettuce shows a better growth performance in hydroponic nutrient solutions based on RAS-derived water (2016). The results of this study let us presume that lettuce growth might still outperform hydroponic growth rates under chemically suboptimal (i.e. high sodium chloride) conditions. However, this study does not explain this phenomenon. A possible explanation could be the beneficial interaction between microorganisms from the RAS water and the plant roots. Earlier, Mayak et al. (2004) described conferred stress resistance in tomato against sodium due to symbiosis with bacteria. With a larger setup and uniform cultivation conditions, this study does not falsify earlier findings by Delaide et al. (2016) that integration between fish production and plant cultivation—aquaponics—could lead to stronger plant development. Further studies are currently carried out in order to validate this hypothesis. Future studies should also clarify whether similar conclusions could be drawn, if the same amount of sodium chloride as in the RAS-derived water were added to the hydroponic group, or studies where RAS-derived water was sterilised before supplying it to the root zone.
